# Papular granuloma annulare of palms and soles: case report of a rare presentation.

**DOI:** 10.12688/f1000research.3-32.v1

**Published:** 2014-01-30

**Authors:** Sidharth Sonthalia, Rahul Arora, Rashmi Sarkar, Uday Khopkar

**Affiliations:** 1 Skinnocence Skin Clinic, Gurgaon, 122009, India; 2 Department of Dermatology and STD, UCMS and Guru Teg Bahadur Hospital, Delhi, 110095, India; 3 Department of Dermatology and STD, Maulana Azad Medical College-Lok N Hospital, New Delhi, 110002, India; 4 Department of Dermatology and STD, Seth G.S. Medical College and King Edward Memorial Hospital, Mumbai, 400012, India

## Abstract

We report the case of a 44-year-old Indian male patient who presented with mildly tender isolated papular lesions confined to the palms of the hands and soles of the feet. The histopathology was characteristic of granuloma annulare. There was an excellent response with 4-week treatment with a potent topical steroid ointment and no recurrence was reported at the follow-up one year later. This report is interesting because of the rare presentation of a common disease.

## Introduction

Granuloma annulare (GA) is a relatively common benign inflammatory disorder characterized clinically by dermal papules and annular plaques. First described by T. Colcott Fox ^1^, it occurs in all age groups and the precise etiology remains unknown. A number of clinical variants have been described in the literature but lesions localized to palms and soles are rare ^1, 2^. Correlation with histopathological findings, including hyperkeratosis, presence of foci of necrobiosis (fragmented collagen), interstial lymphohistiocytic infiltrate in a palisading fashion, and increased mucin deposition, are important for diagnosis of such lesions. We describe a rare case of the papular variant of granuloma annulare with isolated involvement of the palms and soles.

## Case report

A 44-year-old Indian man presented with multiple, slightly painful, raised lesions over the palms and soles that had been present for 3 months. The patient reported having sustained an insect-bite a few weeks prior to the eruption. The patient was a farmer by occupation. His past medical history was insignificant with no history or symptoms suggestive of hypertension, diabetes mellitus, chronic obstructive pulmonary disease or any nutritional deficiencies. He was a non-smoker and reported no relevant family history.

**Figure 1.**
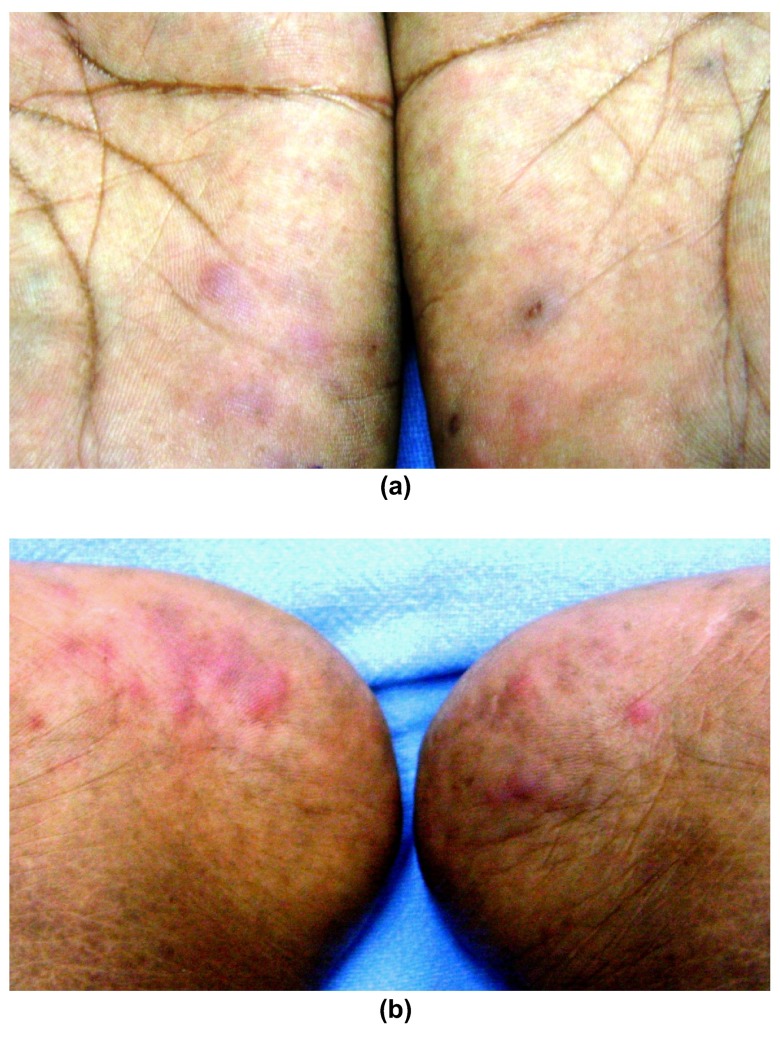
Morphology of cutaneous lesions. Multiple tender dusky red-colored papules and plaques over ( **a**) both palms, and ( **b**) medial aspect of both soles.

**Clinical findings**: Cutaneous examination revealed multiple mildly tender dusky red-colored papules over both palms and the medial aspects of both soles ( Figure 1a and b) with truncal sparing.

**Figure 2.**
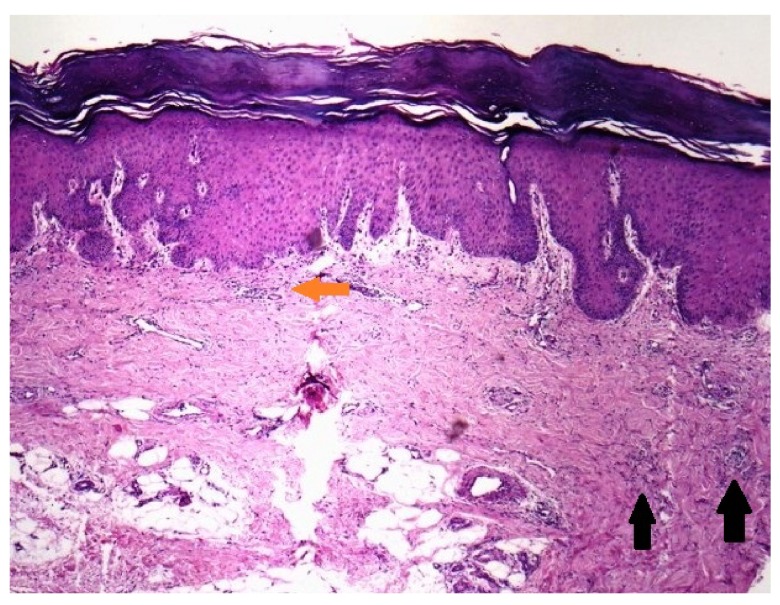
Cutaneous biopsy findings. Histopathology from the palmar lesion revealed hyperkeratosis, acanthosis, superficial and deep perivacular lymphohistiocytic infiltrate (highlighted by red horizontol arrow) with few neutrophils scattered in the reticular dermis, in a background of incomplete collagen degeneration and interspersed mucin (highlighted by two straight black arrows in the left lower aspect). (HE staining, 100×).

**Diagnostic tests**: Hematological and biochemical investigations including a complete hemogram, plasma glucose levels (fasting and post prandial), liver and renal function tests were within normal limits except for a raised Erythrocyte Sedimentation Rate. Serum angiotensin converting enzyme (ACE) levels and serum calcium and phosphate levels we re also normal. Serology for HIV and VDRL tests were negative. Chest radiograph was unremarkable and Mantoux test was negative. Histological examination was done from a 3 mm sized skin biopsy specimen taken from the palm and stained with hematoxylin and eosin (H & E). It revealed hyperkeratosis, acanthosis, superficial as well as deep perivascular mixed infiltrate of lymphocytes, histiocytes and neutrophils in the dermis, over a background of incomplete collagen degeneration, with interspersed mucin ( Figure 2). Based on the clinical and histopathological features, a diagnosis of the papular variant of granuloma annulare localized to the palms and soles was made.

### Treatment and follow up

**Figure 3.**
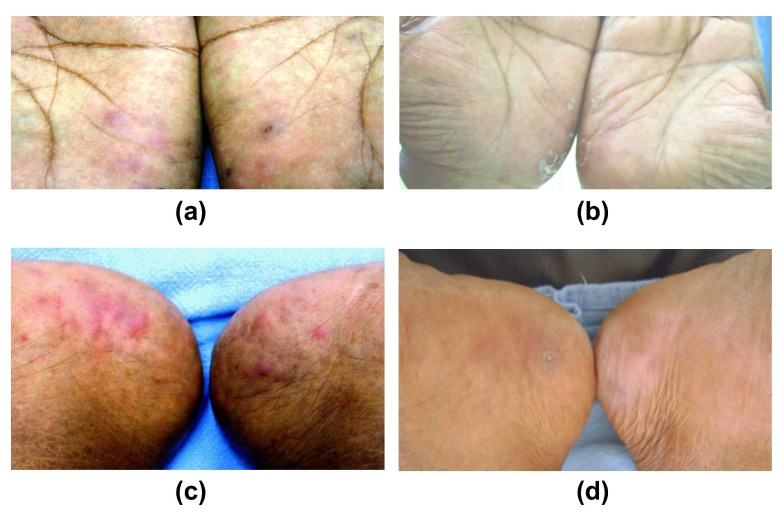
Treatment response. Treatment response with 4 weeks of treatment with potent topical corticosteroid ointment – ( **a**) palmar lesions before treatment, ( **b**) palmar lesions after treatment with almost complete clearance of lesions and mild desquamation, ( **c**) plantar lesions before treatment, and ( **d**) cleared soles with only mild erythema and focal desquamation.

The patient was counselled regarding the benign nature of the disease and started on a twice daily application of a potent topical corticosteroid (clobetasol propionate 0.05%) ointment. This completely resolved the papules within 4 weeks (except for mild post-inflammatory desquamation) with no recurrence reported at the follow up visit one year later ( Figure 3).

## Discussion

GA, a common benign skin condition of unknown etiology has many clinical variants including localized, generalized, papular, umblicated, subcutaneous, perforating, follicular, pustular, and actinic granuloma. The localized variety is the most common and presents with skin-colored to violaceous papules in an annular configuration, most commonly over the dorsal side of the feet, ankles, lower limbs and wrists. In the generalized variety (encountered in only 15% cases), lesions are more widespread, consisting of papules as well as plaques, and involve the trunk, neck, extremities, face, scalp, palms and soles ^3^. Therefore, while palms and soles are frequently involved in the less common generalized variant, they are usually spared in the localized papular variant, which typically affects the dorsa of hands and feet. The subcutaneous variety of GA is characterized by firm asymptomatic nodules in deep subcutaneous tissues, usually over the anterotibial plateau, ankles, dorsa of the feet, buttocks and scalp ^4^. The rare perforating variety presents with superficial umblicated papules with keratotic crusts, usually present over the dorsa of hands and fingers, trunk or extremities ^5^. When GA involves the palms and soles, it may present with tender erythematous annular plaques (usually as a part of generalized variant), subcutaneous nodules or perforating lesions ^6^. In our case, the patient had localized papular GA involving the palms and soles, a rare presentation, with only few cases reported in literature ^6– 11^. The largest case series was a clinicopathological description of seven cases with palmar lesions of GA by Gutte *et al.* ^6^. They reported a palisading and interstitial pattern on histopathology, which is typical of GA lesions elsewhere on the body. Perineural granuloma, perieccrine granuloma and elastophagocytosis with mucin deposition were additional findings in some cases ^6^.

A number of disorders can clinically and histologically mimic GA, including interstitial granulomatous dermatitis with arthritis, palisading neutrophilic and granulomatous dermatitis, rheumatoid nodule and granulomas of Churg Strauss disease ^3^. Erythema elevatum diutinum, acral Sweet’s syndrome, dermatofibroma and drug-induced granulomatous eruptions are other conditions which should be considered in a patient presenting with painful acral papules ^6, 12^.

GA is considered to be an immune-mediated reaction, more specifically, a delayed type IV hypersensitivity response, but the exact etiology remains unknown. Various precipitating factors have been proposed, including trauma, insect bites, viral infections, sun exposure, and medication ^1^. Our patient reported an insect bite that had occurred a few weeks prior to the eruption.

Treatments that have been proposed for GA include topical or intralesional steroids, cryotherapy, electrocoagulation, laser destruction, phototherapy, topical imiquimod, and systemic agents such as antimalarial drugs, corticosteroids, isotretinoin, dapsone, cyclosporine niacinamide, vitamin E, pentoxyfylline and infliximab ^13^. In our patient, treatment with a potent topical corticosteroid (clobetasol propionate 0.05%) ointment resulted in complete resolution in 4 weeks. The patient did not develop lesions elsewhere, nor did he report any recurrences at the one-year follow up. Hence, the patient did not require any systemic drug treatment for his GA to date.

## Consent

Written informed consent for publication of the clinical details, and clinical images, was obtained from the patient.
