# Supplementary material for: Papular granuloma annulare of palms and soles: case report of a rare presentation
Source: F1000Res. 2014 Jan 30;3:32. [Version 1] doi: 10.12688/f1000research.3-32.v1 (PMC3945771; doi:10.12688/f1000research.3-32.v1)
Supplement: Peer review history

# Peer review: Referee response for version 1

**Published:** 2014-02-04

**Authors:** Mohamed Badawy Abdel-Naser

**Affiliations:** 1 Faculty of Medicine, Ain Shams University, Cairo, Egypt

This is a well written and interesting case report. There are few remarks.

 

**Abstract:**

- The phrase “palms and soles” is better than “palms of the hands and soles of the feet”.

**Cutaneous findings:**

-  One of the violaceous palmar papules shows a central umblication! Is it a perforating lesion? Perhaps you can add a little comment on it.

**Diagnostic tests:**

- It was a good to exclude STDs, namely syphilis by serology as tender violaceous papules of palms and soles are important clinical feature of its secondary stage.
   
- The histopathology photo is of insufficient quality and the described findings of GA are difficult to confirm.

**Treatment and follow up:**

- As the authors mentioned, the patient is a farmer. Was it better to apply the potent steroid once at night and under occlusion for better compliance and efficacy?

I have read this submission. I believe that I have an appropriate level of expertise to confirm that it is of an acceptable scientific standard.

# Peer review: Referee response for version 1

**Published:** 2014-01-30

**Authors:** Maxwell Fung

**Affiliations:** 1 Department of Dermatology, University of California, Davis, Sacramento, 95816, USA

The authors have submitted an interesting, relatively novel, and clinically relevant case of acral papular granuloma annulare (GA).

I cannot independently verify the diagnosis of GA based on the photomicrograph provided. An image obtained at higher magnification may be required to clearly demonstrate the granulomatous inflammation and necrobiosis.

In their Discussion, the authors describe the clinical and histologic differential diagnosis for acral GA. It may be helpful to emphasize some relevant clinical aspects, including the association with pain (beyond tenderness) and arthalgia mimicking rheumatologic disease, and a possible correlation with systemic malignancy ( Barksdale *et al.,* 1994). Idiopathic palmoplantar hidradenitis might be reasonably considered in the clinical differential diagnosis; in this regard, since neutrophils were present in the reticular dermis in this case, it may be helpful to document that eccrine glands were uninvolved to eliminate this possibility from consideration.

As for any case report describing treatment of a disorder whose natural history is to wax and wane, it is uncertain whether the relationship between treatment and clinical improvement is causation versus mere correlation.

I have read this submission. I believe that I have an appropriate level of expertise to confirm that it is of an acceptable scientific standard.
